# Combination of DCE-MRI and DWI in Predicting the Treatment Effect of Concurrent Chemoradiotherapy in Esophageal Carcinoma

**DOI:** 10.1155/2020/2576563

**Published:** 2020-06-16

**Authors:** Changmin Liu, Roger Sun, Jing Wang, Fangling Ning, Zhenbo Wang, Judong Luo, Shaoshui Chen, Shuanghu Yuan

**Affiliations:** ^1^Department of Oncology, Binzhou Medical University Hospital, The Yellow River Two Road No. 661, Binzhou, 256603 Shandong, China; ^2^Department of Radiation Oncology, Gustave Roussy Cancer Campus, 94800 Villejuif, France; ^3^Department of Radiology, Binzhou Medical University Hospital, The Yellow River Two Road No. 661, Binzhou, 256603 Shandong, China; ^4^Department of Radiotherapy, Changzhou Second People's Hospital, Affiliated to Nanjing Medical University, The Xinglong Xiang, No. 29, Changzhou, 213000 Jiangsu, China; ^5^Department of Radiology, Shandong Cancer Hospital and Institute, Shandong First Medical University, Jiyan Road No. 440, Jinan, 250117 Shandong, China; ^6^Shandong Cancer Hospital and Institute-Shandong Cancer Hospital Affiliated to Shandong University, Jiyan Road No. 440, Jinan, 250117 Shandong, China

## Abstract

**Background:**

Concurrent chemoradiotherapy (CCRT) is the main treatment for esophageal cancer, but the response to treatment varies from individual to individual. MR imaging methods, such as diffusion-weighted (DW) MRI and the use of dynamic contrast-enhanced (DCE) MRI, have the potential to provide additional biomarkers that could evaluate the effect of CCRT in patients with esophageal carcinoma.

**Materials and Methods:**

Fifty-six patients with esophageal carcinoma, verified by histopathology, underwent MRI examination before and at midtreatment (4^th^ week, radiotherapy 30–40 Gy) using the Siemens 3.0 T MR System. Parameter maps of apparent diffusion coefficient (ADC), and DCE maps of volume transfer constant (*K*^rans^), rate contrast (*k*_ep_), and extracellular fluid space (*v*_e_), were computed using a Siemens Company Multimodality Workplace (MMWP) model. Comparison of histogram parameters and their diagnostic performance was determined using the Mann–Whitney *U* test and receiver operating characteristic (ROC) analysis.

**Results:**

56 patient MRI scans were available for analysis at baseline and at the third week, respectively. Pretreatment *K*^rans^, pretreatment *k*_ep_, pretreatment ADC (*P* < 0.05), and during-treatment *K*^rans^ (*P* < 0.05) and Δ*K*^rans^ and *Δ*ADC (*P* < 0.05) were significantly different after CCRT. Based on the binary logistic model, the ROC analysis demonstrated that the combined predictors demonstrated a high diagnostic performance with an AUC of 0.939. The sensitivity and specificity were 98.6% and 73.8%, respectively.

**Conclusion:**

The combination of DCE and DWI can be used as an early biomarker in the prediction of the effect of CCRT three weeks after treatment in esophageal carcinoma.

## 1. Background

CCRT is a primary treatment method for esophageal carcinoma. However, curative effect of this modality considerably varies among different individuals. Thus, an effective means for individualized prediction of the efficacy of such method is urgently needed. With the development of functional imaging technologies, diffusion-weighted imaging (DWI) has been used to evaluate esophageal carcinoma, but its clinical applications retain many limitations [1, 2]. DCE can rapidly evaluate the status of tissue microcirculation through imaging. Currently, DCE has shown to be effective for tumor diagnosis, tumor grading, tumor response evaluation, and recurrence prediction, for several cancer types such as breast cancer [3–5], liver cancer [6, 7], rectal cancer [8, 9], pancreatic cancer [10], nasopharyngeal carcinoma [11, 12], and prostate cancer [13, 14].

## 2. Aim

In this context, with relatively few reports on the application of DCE, this project is aimed at analyzing the efficacy of combining this modality with DWI in evaluating CCRT for esophageal carcinoma.

## 3. Materials and Methods

### 3.1. Patients

All patients with histologically proven esophageal epidermoid carcinoma diagnosed in our center from December 1, 2014, to December 1, 2016, were screened for inclusion. Pathology specimens were obtained by gastroscopy. Inclusion criteria were tumors of the cervical esophagus, or inoperable tumors of thoracic esophagus (unfit patients or refusal), patient's Eastern Cooperative Oncology Group (ECOG) score of 0 to 1 and life expectancy > 6 months, and no contraindications for contrast-enhanced MRI. Patients had no esophageal surgery, radiotherapy, or chemotherapy before the study. This retrospective study was approved by the Ethical Committee and done in accordance with the ethical standards of the 1964 Helsinki Declaration and its later amendments. All patients provided informed consents.

### 3.2. MRI Scanning and Image Processing

All patients underwent MRI examination before and at midtreatment (4^th^ week, radiotherapy 30–40 Gy) using the Siemens 3.0 T MR System. Respiratory gating and respiratory triggering techniques were used during the MR imaging acquisitions.

Multiple body position scanning (transverse section, median sagittal section, and coronal section) was employed. First, spin-echo sequence was swept, and then, median sagittal section T1-weighted imaging (TIWI), transverse-section TIWI, and T2WI lipid sequence scans were performed. DWI sequences use short-time inversion to restore echo-planar imaging sequence. Scanning parameters were as follows: repetition time (TR) = 5.3 s, echo time (TE) = 56 ms, 24 layers with 5.0 mm thickness, layer spacing = 0.5 mm, field of view (FOV) = 314 mm × 380 mm, scan matrix = 256 × 256, number of NEX = 2, asset (array spatial sensitivity encoding technique) value = 2.0; and free breath. Scanning range included all tumors (upper bound to thyrocricoid and lower bound to upper renal margin), and scanning time was 64 s. Diffusion sensitivity coefficient *b* was 500 s/mm^2^. When the ADC value is more than 3 times of the average value in the same period, we judged that the deviation was large and will be eliminated.

DCE scan sequence employs a three-dimensional VIBE for the median sagittal section. Scan parameters were as follows: TR = 5 s, TE = 2 ms, slice thickness = 1.5 mm, FOV = 360 mm × 225 mm, and 35 periods in total. A total of 18 slices were acquired per sequence, leading to a total of 630 slices. Each scan period lasted for 5.9 s and repeated for 20 measurements. The following steps were applied for the prescan. Before injecting the contrast agent, a scan was performed to obtain the mask. Then, 0.1 mmol/kg of contrast agent gadolinium diamine was applied based on weight and injected at 2 ml/s. During the scanning interval, the contrast agent was injected, and a multiple-phase scan was started. Patients underwent breathing training before undergoing MRI scans. The scan was conducted after breath hold.

**Figure 1:**
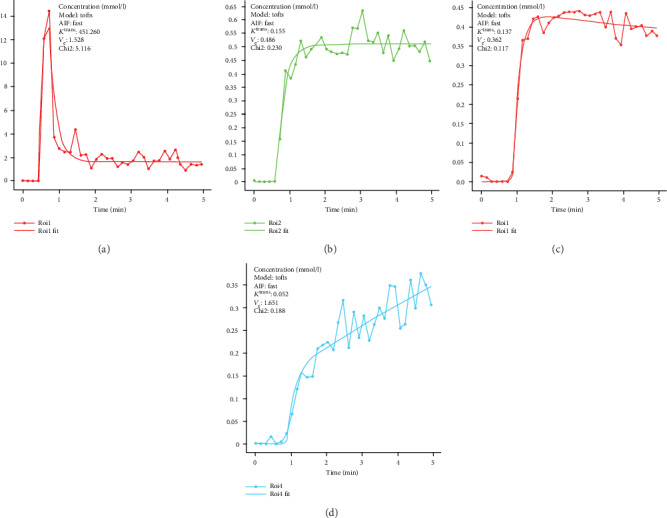
TIC type. (a) Type I; (b) type II; (c) type III; (d) type IV.

The image was processed by the Siemens Company MMWP. Initially, a rigid fusion before and after MRI was required. When an anatomic mark was difficult to match, findings were registered according to the esophageal image. To achieve this goal, the first MRI image obtained before treatment was considered the main image reference, whereas the MRI image in treatment metaphase was assigned as the secondary image reference. Region of interest (ROI) was required to manually trim the surrounding normal organization. Then, ADC value and DCE image parameters were measured. These parameters included *K*^rans^, *k*_ep_, and *v*_e_. The maximum diameter level of tumor in the DCE image was then selected for ROI construction, and signal strength curve type was measured (time of intensity curve, TIC). TICs were divided into the following four types depending on peak time and contrast agent outflow in the delay phase [15, 16], type I (fast-rising–descending type), type II (fast-rising–platform type), type III (slow-rising–platform type), and type IV (continuous-rising type) (Figure 1). In this study, the maximum diameter level of tumor was retrieved, and types of TIC curve recorded before CCRT were compared.

An ROI of the normal esophagus was also defined at the same level than the tumor as a control for the different parameters. The delta-parameters, corresponding to the difference of the parameter's values between the midtreatment and the baseline MRI, were also computed.

### 3.3. Treatment

Radiotherapy was delivered using three-dimensional conformal radiation therapy (3D-CRT) or intensity-modulated radiation therapy (IMRT) techniques, with a total tumor dose of 59.4–60 Gy (1.8–2.0 Gy/d, 5 f/w). The esophageal gross tumor volume (GTVt) and nodal GTV (GTVn) (regional lymphatic metastasis) were defined according to the results of a CT scan (from cartilage to the lower edge of the liver) and gastroscopy inspection. CTVt: GTVt expands 3.0 cm at the upper and lower ends and 0.5 cm at the front, back, left, and right. CTVn: 0.5 cm outside in all directions of GTVn. The anatomical barrier needs to be adjusted after it is placed. For PTV, CTV expands uniformly by 0.5 cm, left and right, and 0.5-0.8 cm. Weekly cone-beam computed tomography validation was performed to ensure a good patient repositioning. Concomitant chemotherapy regimen used FP (cisplatin+5-fluorouracil), or cisplatin+tegafur regimen. Chemotherapy was performed at the first and fourth weeks after radiotherapy.

### 3.4. Therapeutic Evaluation

Clinical outcomes were evaluated on a CT scan within one week at the end of radiotherapy and were defined as complete response (CR), partial response (PR), stable disease (SD), and progressive disease (PD) according to the Response Evaluation Criteria in Solid Tumors version 1.1. An increase of the diameter of the tumor by at least 5 mm and the occurrence of one or more new lesions were also considered as a PD.

### 3.5. Statistical Analysis

Statistical analysis was performed using SPSS 20.0 software. The Mann–Whitney *U* test or *t*-test was used for numerical variables. The Chi-square test was used for categorical variable. The receiver operating characteristic (ROC) curve was used to calculate for parameters and predict the effectiveness of parameter combinations. A threshold *P* value of less than 0.05 was defined as significant in two-tailed analyses.

## 4. Results

Seventy-six patients were available for screening. After screening, a total of 56 patients were included and evaluable in the final analysis (8 failed to complete MRI inspection, and 7 were excluded because of the failure of MRI image to meet determination requirements). Patients were predominantly male (*n* = 47 (84%)) with a median age of 67.2 years (range = 52–80 years). The tumor type was squamous carcinoma for 55 patients (98%) and adenocarcinoma for one patient. Characteristics of patients are summarized in Table 1.

### 4.1. Recent Therapeutic Evaluation

Baseline tumor volume ranged from 3.20 cm^3^ to 76.29 cm^3^ (average: 35.30 ± 20.59 cm^3^). At midtreatment, we again measured tumor volume, ranging from 2.014 to 47.456 cm^3^ (average: 22.479 ± 13.349 cm^3^). Tumor regression rate during the first phase of the treatment totaled 0%–76.21% (average: 51.64%). According to the CT scan within one week at the end of radiotherapy, 14 patients (25%) were classified into the CR group, whereas 42 cases were assigned to the non-CR group (SD+PR+PD with 5, 37, and 0 cases, respectively) (85.0%). No significant differences were identified regarding clinical factors (sex, age, clinical staging, N stage, pathologic type, and lesion site) between the two groups (*P* > 0.05) with the exception of T stages (*P* < 0.05) (Table 1).

### 4.2. DWI Performance

**Figure 2:**
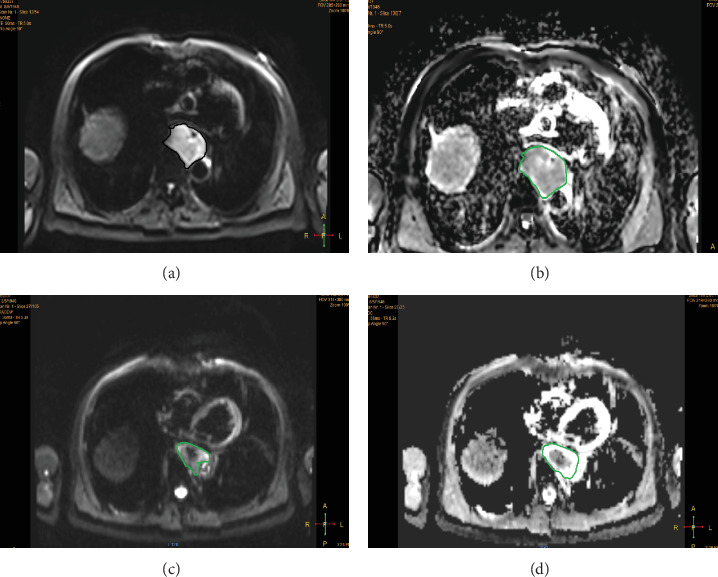
DWI performance before and mid-CCRT. (a, b) DWI and ADC map graphs, respectively, before treatment showed that lesions in the esophagus exhibited a significantly high signal; average esophageal lesions were measured with an ADC value of 2.371 × 10^−3^ mm^2^/s. (c, d) In DWI and ADC map graphs for mid-CCRT, high signal range of esophageal lesions was significantly diminished. Signal intensity was altered, and average ADC value measured 3.015 × 10^−3^ mm^2^/s.

At baseline, tumor showed high signal intensity on DWI for 55 out of 56 patients (98.21%). At midtreatment, high signal intensity on DWI sequences remained for 43 patients, and the mean ± standard deviation of ADC values (×10^−3^ mm^2^/s) and GTV volumes (cm^3^) based on the 3D map at the baseline and midtreatment points were 1.733 ± 0.517, 2.506 ± 0.488 and 35.30 ± 20.59, 22.479 ± 13.349, respectively. This shows that the ADC values increased gradually along with the increased radiation doses (Figure 2).

### 4.3. Relationship between DCE TIC Type and Therapeutic Response

At baseline, the TIC of tumors showed a majority of types I and II (*n* = 44/56 (78.6%)), while at midtreatment, TICs were predominantly of types III and IV (*n* = 37/56 (66.1%)). Regarding the TIC of the control ROIs interesting the normal esophagus, there were a majority of types III and IV, at both baseline and midtreatment: *n* = 46/56 (82.1%) and *n* = 48/56 (85.7) (Table 2).

### 4.4. Changes in DCE Parameters and ADC Values before and Mid-CCRT

**Figure 3:**
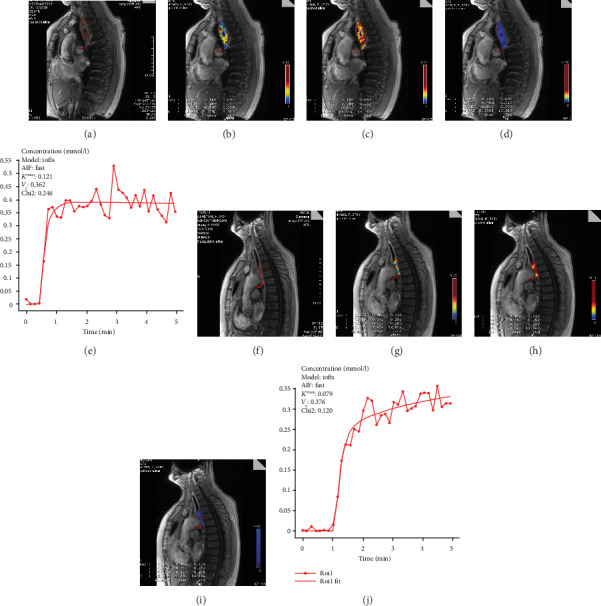
Changes in DCE parameters and image before and mid-CCRT. Before CCRT (a–e): maximum-level ROI schematic, *K*^rans^, *v*_e_, *k*_ep_, and TIC type of tumor in before treatment. Mid-CCRT (f–j): maximum-level ROI schematic, *K*^rans^, *v*_e_, *k*_ep_, and TIC type of tumor in mid-CCRT.

DCE quantitative parameters (*K*^rans^, *k*_ep_, and *v*_e_) and DWI parameter ADC values for the ROIs of tumor and normal esophagus at baseline and mid-CCRT are detailed in Table 3 and were analyzed by paired methodology. For the tumor ROI, there were significant differences in *K*^rans^, *v*_e_, and ADC (*P* < 0.05) but no significant difference in *k*_ep_ (*P* > 0.05) between baseline and mid-CCRT. For the normal esophagus, there were no significant differences regarding *K*^rans^, *v*_e_, and ADC (*P* < 0.05) (Table 3) between baseline and mid-CCRT MRI parameters. ROI illustration and parameter images are shown in Figure 3.

Results of DCE-MRI and DWI parameter of the tumor ROI (at baseline and mid-CCRT) between the CR and non-CR groups are detailed in Table 3. At baseline, pre-*K*^rans^, pre-*k*_ep_, and ADC were all significantly higher in the CR group. Only the pre-*v*_e_ was not significantly differed between the two groups (*Z* = −1.139, *P* = 0.255). Regarding the mid-CCRT MRI, only the mid-CCRT-*k*_ep_ was significantly different between the two groups (0.323 ± 0.140/min for the CR group and 0.373 ± 0.113/min for the non-CR group, *Z* = −2.394, *P* = 0.017) (Table 4).

### 4.5. The Relationship between the Variation of DCE, DWI Parameters, and Curative Effect

Results of the association between the clinical response and the variation of the MRI parameter's value (delta-parameters) between baseline and mid-CCRT are presented in Table 5. Results showed Δ*K*^rans^ values of 0.046 ± 0.090 and 0.025 ± 0.088 for the CR and non-CR groups, respectively; these values significantly differed between the two groups (*Z* = 0.841, *P* = 0.037). *Δ*ADC values amounted to 0.805 ± 0.689 and 1.029 ± 0.633, respectively, and significantly differed between the two groups (*Z* = 5.133, *P* = 0.029) (Table 5).

### 4.6. Performance of DCE ROC Parameters in Calculating for the Predicted Curative Effect of Each Parameter

**Figure 4:**
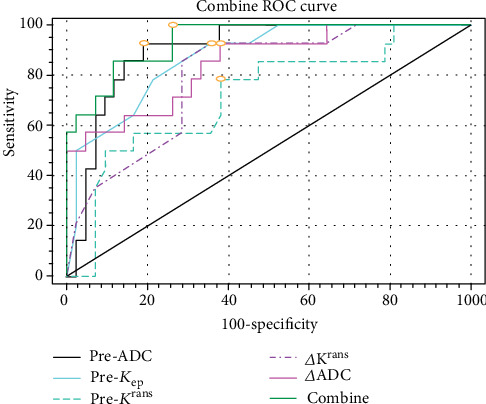
ROC curves of parameters. The joint factor (Combine) area under the ROC curve equaled to 0.939, with sensitivity of 98.6%, specificity of 73.8%, and Youden index of >0.211, which is superior to single sequence in CCRT for esophageal cancer.

To determine the cut point of DCE and DWI parameters over the baseline and midtreatment used for “CR” vs. “PR,” one ROC analysis was conducted. The results are shown in Table 6. The analysis indicated that the area under the curve for pre-ADC values was the biggest; the values were 0.901, and the sensitivity and specificity were 91.6% and 81.0%. Pre-*K*^rans^, pre-*k*_ep_, pre-ADC, Δ*K*^rans^, and *Δ*ADC were inputted into joint detection equation (Combine), and ROC analysis was conducted. The result showed that the area under the ROC curve of joint factor equaled to 0.939, with sensitivity of 98.6%, specificity of 73.8%, and Youden index of >0.211 (Figure 4).

## 5. Discussion

DCE-MRI is a functional imaging method for noninvasive evaluation of tissues and pathological microcirculation characteristics; this method has been widely used in differential diagnosis of multiple tumors, evaluation of curative effect, and prediction of recurrence in cancers, such as breast cancer [17, 18], liver cancer [19–21], ovarian cancer [22, 23], head-and-neck tumors [24, 25], and pancreatic cancer [26, 27]. Along with the support from the research department of Siemens, we designed a VIBE sequence for esophageal tumor. The group also analyzed changes in parameters before and mid-CCRT by the Tofts model and combined the process with DWI technology. These steps were conducted to screen out a prediction method that is noninvasive, dynamic, and suitable for clinical promotion.

By the DCE-MRI scan, all cases can be observed in the esophageal lesion site. The scan also revealed degree of development of tumor tissue in each layer and the relationship of this tumor with surrounding tissues. Pseudocolor map of each parameter shows lesion boundaries and provides a new reference for determining GTV of tumor target areas. Regarding assessment of perforations or obstructions by tumor, the proposed method holds clear and stereoscopic advantages over CT scan, barium meal, and DWI. Constructing a TIC curve also allowed observation of significant differences between types of esophageal cancer tissues and the normal esophageal wall. Esophageal carcinoma tissue mainly showed TIC types I (fast-rising–descending type) and II (fast-rising–platform type), whereas normal esophageal wall chiefly revealed types III (slow-rising–platform type) and IV (continuous-rising type). Mid-CCRT, TIC tumor tissue types were differentiated into types III and IV, which were absent in the normal esophageal canal wall (control). These observations coincided with those of TIC types in other tumors detected by DCE-MRI [28–30]. This result was achieved probably because radiation damage from radiotherapy caused tumor capillary wall swelling, degeneration, necrosis, luminal narrowing, and thrombosis. These occurrences then resulted in decreased blood flow perfusion, diminished blood vessel leakage, and localized fibrosis, or the presence of ordering, thus leading to conversion of TIC type into a normal state.

In this study, DCE-MRI can reflect external invasion of esophageal lesions, degree of esophageal stenosis, and the relationship with surrounding tissues. However, imaging modality holds some drawbacks; the most prominent of which is the inability of scanning mode to reflect mediastinal lymphatic metastasis in an omnidirectional pattern. To compensate for this insufficiency, we also scanned DWI sequence and observed esophageal lesions and mediastinal lymphatic metastatic foci through high-metabolism images. By observing changes in DWI and DCE-MRI parameters *K*^rans^, *k*_ep_, *v*_e_, and ADC in different periods, we noted that DWI and DCE-MRI parameters were related to tumor regression. Lei et al. [31] compared 25 cases of esophageal cancer patients through a predictive effect of DCE-MRI and reached the same conclusion. However, some other studies showed results that are inconsistent with conclusion of the present project [32, 33]; thus, causes of these differing results were analyzed for differences. On the one hand, in the ROI schematic used in this project, median sagittal section of the tumor area in each layer was set to the ROI to reduce errors. Final calculation of averages then diminished errors caused by different ROIs in different periods. On the other hand, various studies presented differences in MRI scan sequences. We assumed that DWI and DCE can jointly predict synchronous CCRT, and the relationship between lesions and surrounding tissues was demonstrated by multidimensional degrees.

In conclusion, as a noninvasive functional imaging technology, DCE-MRI can reflect changes in tumor tissue structure, biochemical metabolism, and microenvironment during occurrence and progression of esophageal carcinoma and CCRT. The modality also helps determine tissue response to treatment in the early stage and provides basis for adjustment of follow-up treatment plan. As a new technology for esophageal carcinoma application, various aspects, such as scanning posture, analysis model, and appropriate inversion angle, must be explored. However, combination of DCE-MRI with DWI sequence still suggests the former's high clinical application value.

## Figures and Tables

**Table 1:** Patient response and clinicopathological characteristics.

Characteristics	Cases (%)	Response		Chi-square test *P* value
		CR (*n*)	Non-CR (*n*)	
*Gender*				
Male	47 (83.9)	12	35	0.044
Female	9 (16.1)	2	7	0.834
*Age* (*years*)				
≤60	12 (21.4)	4	8	0.566
>60	44 (78.6)	10	34	0.452
*Clinical stage*				
I	14 (25.0)	6	8	3.508
II	22 (39.3)	5	17	0.173
III	20 (35.7)	3	17	
*T classification*				
T_1_	4 (7.1)	3	1	8.000
T_2_	24 (42.9)	6	18	0.046
T_3_	20 (35.7)	5	15	
T_4_	8 (14.3)	0	8	
*N classification*				
N_0_	24 (42.9)	6	18	0.000
N_1_	16 (28.6)	4	12	1.000
N_2_	16 (28.6)	4	12	
*Pathological type*				
Squamous cell carcinoma	55 (98.2)	0	1	0.339
Adenocarcinoma	1 (1.8)	14	41	0.560
*Location of primary tumor*				
Ce	6 (10.7)	3	3	4.848
Ut	22 (39.3)	3	19	0.183
Mt	24 (42.9)	6	18	
Lt	4 (7.1)	2	2	

Ce: cervical esophagus; Ut: upper thoracic esophagus; Mt: middle thoracic esophagus; Lt: lower thoracic esophagus.

**Table 2:** Comparison of TIC types of before and mid-CCRT.

		Type 1	Type 2	Type 3	Type 4	*P* value
Tumor	Baseline	19 (33.9%)	25 (44.6%)	4 (7.1%)	8 (14.3%)	<0.001
	Mid-CCRT	4 (7.1%)	15 (26.8%)	25 (44.6%)	12 (21.4%)	
Control	Baseline	3 (5.4%)	7 (12.5%)	14 (25.0%)	32 (57.1%)	0.383
	Mid-CCRT	3 (5.4%)	5 (8.9%)	12 (21.4%)	36 (64.3%)	

**Table 3:** Comparison of parameters of before and mid-CCRT (mean ± sd).

Parameter	Tumor ROI			Normal tissue ROI		
	Pre-CCRT	Mid-CCRT	*P* value	Pre-CCRT	Mid-CCRT	*P* value
DCE						
*K*^rans^	0.169 ± 0.076	0.128 ± 0.036	0.001	0.089 ± 0.028	0.085 ± 0.046	0.077
*k*_ep_	0.399 ± 0.139	0.361 ± 0.121	0.097	0.285 ± 0.067	0.265 ± 0.072	0.102
*v*_e_	0.466 ± 0.137	0.372 ± 0.091	0.002	0.394 ± 0.072	0.365 ± 0.199	0.065
DWI(×10^−3^ mm^2^/s)						
ADC	1.733 ± 0.517	2.506 ± 0.488	<0.001	3.316 ± 0.638	3.501 ± 0.719	0.057

**Table 4:** The parameter of tumor ROI in different therapeutic groups (mean ± sd).

Parameter	Pre-CCRT			Mid-CCRT (4^th^ week)		
	CR	Non-CR	*P* value	CR	Non-CR	*P* value
DCE						
*K*^rans^	0.173 ± 0.019	0.167 ± 0.078	0.012	0.149 ± 0.033	0.121 ± 0.035	0.803
*k*_ep_	0.261 ± 0.113	0.416 ± 0.082	0.000	0.323 ± 0.140	0.373 ± 0.113	0.017
*v*_e_	0.494 ± 0.092	0.457 ± 0.149	0.255	0.376 ± 0.156	0.368 ± 0.150	0.323
DWI(×10^−3^ mm^2^/s)						
ADC	2.282 ± 0.943	1.551 ± 0.658	0.000	2.286 ± 0.532	2.580 ± 0.456	0.058

**Table 5:** The parameter of tumor ROI change of different therapeutic groups (mean ± sd).

Parameter	CR	Non-CR	*Z*	*P* value
DCE				
Δ*K*^rans^	0.046 ± 0.090	0.025 ± 0.088	0.841	0.037
Δ*k*_ep_	0.038 ± 0.132	−0.063 ± 0.190	-1.514	0.130
Δ*v*_e_	0.094 ± 0.145	0.095 ± 0.189	-0.684	0.494
DWI(×10^−3^ mm^2^/s)				
*Δ*ADC	0.805 ± 0.689	1.029 ± 0.633	5.133	0.029

**Table 6:** The results of ROC curve analysis.

	Area under curve	Sensitivity (%)	Specificity (%)
Pre-*K*^rans^	0.721	78.6	60.6
Pre-*k*_ep_	0.874	92.9	64.3
Pre-ADC	0.901	91.6	81.0
Δ*K*^rans^	0.799	92.9	62.5
*Δ*ADC	0.849	92.1	60.8

## Data Availability

The data used to support the findings of this study are available from the corresponding author upon request.
